# Quasi-stabilized hydration layers on muscovite mica under a thin water film grown from humid air

**DOI:** 10.1038/s41598-017-04376-3

**Published:** 2017-06-22

**Authors:** Toyoko Arai, Kohei Sato, Asuka Iida, Masahiko Tomitori

**Affiliations:** 1 Graduate School of Natural Science and Technology, Kanazawa University, Kanazawa, Ishikawa 920-1192 Japan; 2 School of Materials Science, Japan Advanced Institute of Science and Technology, Nomi, Ishikawa 923-1292 Japan

## Abstract

The interfaces between solids and water films in air play fundamental roles in physicochemical phenomena, biological functions, and nano-fabrication. Though the properties of the interfaces have been considered to be irrelevant to the water film thickness, we found distinctive mechanical features of the interface between a cleaved muscovite mica surface and a thin water film grown in humid air, dissimilar to those in bulk water, using frequency-modulation atomic force microscopy. The thin water film grew with quasi-stabilized hydration networks of water molecules, tightly bound each other at the interface, to a thickness of ~2 nm at near-saturating humidity. Consequently, defective structures of the hydration networks persisted vertically through the hydration layers at the interface, and K^+^ ions on the cleaved surface remained without dissolution into the water film. The results provide atomistic insights into thin water films in regard to epitaxial-like growth from vapour and the motion of water molecules and ions therein.

## Introduction

Most solid surfaces are covered with a water film under ambient conditions^1–4^. Water films on these surfaces have crucial roles as either promoters or inhibitors in processes such as wetting, deliquescence, corrosion, catalysis, colloid, friction, and weathering. The water film is divided into three parts: a solid–water interface, a water layer, and a water–vapour interface. Understanding of the chemical structure and associated properies of each part is of great importance to control the phenomena and apply them to industrial products operated under ambient conditions.

On solid surfaces under water, the solid–water interface takes a central role for chemical reactivity, whereas the water layer dominantly acts as a supplier or a receiver for the mass transportation. For a thin water film on a solid surface in air, its water layer is very thin or none, and the water–vapour interface is very close or overlapped with the solid–water interface. Consequently, the characteristics of the solid–water interface would be altered in the thin water film. However, little is known about the solid–water interface in thin water films. Exploring the structure and properties of the solid–water interface in the thin water film in air is intriguing and challenging for basic science and its industrial applications.

In the study of solid–water interfaces, oxide surfaces have been often used as a model of solid substrate owing to their stability in air. Particularly, muscovite (KAl_2_(Si_3_Al)O_10_(OH)_2_) has been most often used, beneficially to the study, because an atomically-flat wide surface can be easily obtained by cleaving along the (001) plane, which allows analysis with an atomic point of view^1, 2, 4–7^. Muscovite is a representative mineral of the mica group, alternately composed of negatively-charged aluminosilicate layers and K^+^ layers, parallel to the (001) plane. On the top of aluminosilicate layer, a honeycomb lattice of a SiO_2_ tetrahedron sheet exists. The cleavage occurs along the K^+^ layer between the two SiO_2_ tetrahedron sheets. Each K^+^ ion usually remains on either tetrahedron sheet at a fifty-fifty rate after cleavage along the K^+^ layer^8–11^.

When muscovite is cleaved in air, water molecules start to adsorb on the cleaved surface. The water coverage depends on the humidity and temperature of ambient conditions^1–5, 12–14^. Two-dimensional water islands grow at relative humidities (RH) of less than 50%^13^, and a monolayer of water molecules is formed in the range of RH of 50–75%^1, 2, 5, 13–17^. Molecular dynamics (MD) simulations have revealed that the water monolayer exhibits an ice-like structure through the hydrogen bonds between the H atoms of water and the O atoms on the cleaved surface, where the K^+^ ions were surrounded by hydrogen-bonded water cages^16^. At higher RHs, the second water layer grew as two-dimensional islands on the first layer^14^. The height of each layer was 0.37 nm, in agreement with a height of a monolayer of ice-I_h_ (ice-phase-one), and its growth mode was presumed as Frank-van der Merwe mode (layer-by-layer epitaxy)^14^. With increasing RH to 100%, the water film thickness increased up to 2–2.5 nm at room temperature^1, 12^. The behaviour of water layers in the films with thicknesses over three monolayers would be the same with those of bulk water, because the thermodynamic parameters of the water layers were calculated to be close to those of bulk water^5^.

However, there has been no direct experimental evidence to support these theoretical studies. Concerning the mass transportation in the thin water layers, the mobilities of ions and molecules would be low^10, 18, 19^, but they have not been characterized apparently. Accordingly, there have remained questions of how the properties of the thin water films under ambient conditions are in reaction and mass transportation, and whether the ice-like water structure on the muscovite surface under the thin water film remains or not. It is noted that an MD simulation for a 3 nm-thick water film on the muscovite surface suggested the hydration layers with partically-ordered structures, which were neither ice-like nor liquid-like^20^.

The structures of solid–water interfaces on the cleaved muscovite surface immersed in bulk water have been intensively examined^21–33^. It has been ascertained that the water molecules at the solid–water interface on the muscovite surface in bulk water are weakly bound each other to form a network as two-dimensional layers, parallel to the substrate^23–26, 34–36^. The network structure is disordered in comparison with the ice-like structure of one monolayer adsorption of water molecules. The water molecules and ions are likely exchangeable through the hydration layers, which are regarded as liquid-like^1, 23, 34^. The K^+^ ions, remained on the cleaved surface, are easily dissolved into bulk water through the hydration layers^8–10^. Afterwards, the cavity centres left by the K^+^ ions in the honeycomb lattice are then filled with H_2_O or H_3_O^+^, corresponding to the adsorption layer (the layer nearest to the muscovite surface). That is, most K^+^ ions do not exist on the cleaved surface in bulk water^9, 10, 23^. For the thin water films, however, the phenomena related to dissolution can be changed.

Currently, three-dimensional atomic-scale imaging by frequency modulation atomic force microscopy (FM-AFM)^29–31^ and amplitude modulation AFM (AM-AFM)^32, 33, 37^ have been applied to analysis of ions on the muscovite surfaces in bulk water and the hydration layers at their solid–water interfaces. Therein, an AFM cantilever, having a tip at its end, was used as a force sensor immersed whole in bulk water. It was reported that the density of water molecules of the first hydration layer was higher over the honeycomb lattice of the SiO_2_ tetrahedron sheet of muscovite, and the density of the second hydration layer was higher at the cavity centres of the honeycomb lattice, resulted in a honeycomb pattern and a dot-arrayed pattern of the AFM image contrast, respectively. The first and the second hydration layers are referred to as in order of proximity to the muscovite surface, next to the adsorption layer. The third hydration layer, however, has not been imaged, which might be broadened due to a weakly-bound water network. Recently, for the solid–water interfaces in nanometre-thick water films, we have modified the experimental scheme using the FM-AFM setup operated in air; we immersed only the tip apex into the thin water film, and successfully achieved atomic resolution for the hydration layers on a cleaved KBr surface covered with a thin water film^38^. Close examination of the third hydration layer by our experimental scheme would provide valuable information to precisely evaluate the strength of the network between individual water molecules.

Here, we report the FM-AFM observation for a thin water film on a cleaved muscovite surface in humid air. Thin water films with a thickness of 2 nm were grown on the surface from water vapour at near-saturating humidity. The three-dimensional, atomic-scale structure of the solid–water interface was revealed to differ from the structure at the interface between muscovite and bulk water, using the FM-AFM operated in air with only a tip apex immersed in the thin water film. We found that K^+^ ions on the cleaved muscovite surface remained; the ions were not dissolved into the thin water layer possibly. In addition, defective structures of the water network at the interface persisted from the first hydration layer to the second hydration layer. Furthermore, we first observed the lateral honeycomb pattern of the third hydration layer, located densely near the muscovite surface in comparison with the third hydration layer in bulk water. These indicate that the thin water film from water vapour grows epitaxial-like and preserves the features of the cleaved muscovite surface through its tightly-bound quasi-stabilized water network at the solid–water interface, and water molecules and ions move slowly in the thin film.

## Results

### Scheme of FM-AFM for a thin water film on a cleaved muscovite surface in air

**Figure 1:**
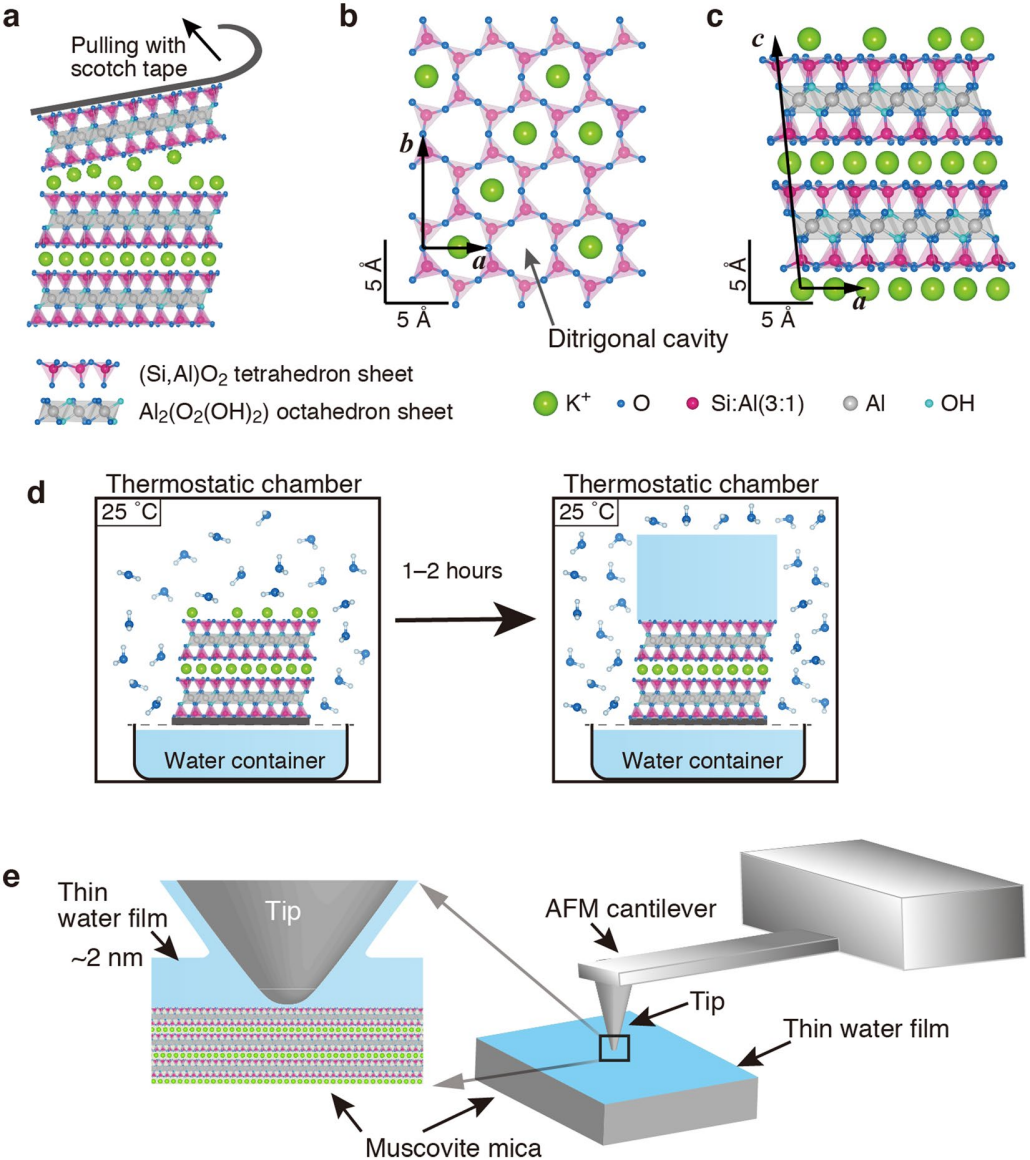
Schematics of preparation of a thin water film on muscovite mica and FM-AFM imaging of the water film. (**a**) Cleavage of muscovite mica along the (001) plane using Scotch tape under ambient laboratory conditions. The cleavage occurs along the K^+^ layer between the two SiO_2_ tetrahedron sheets. Each K^+^ ion usually remains on either tetrahedron sheet at a fifty-fifty rate after cleavage. (**b**) Atomistic model of the cleaved muscovite mica (001) surface. The (001) surface composed of a hexagonal network of (Si,Al)O_2_ and K^+^ ions located over the ditrigonal cavities of the network. (**c**) Side view of the [010]-directional projection. Adjoining two rows of K^+^ ions, running in the [100] direction, on the top of (001) surface are depicted in the projection. K^+^ ions, O atoms, Si atoms, Al atoms, and OH groups are shown in green, blue, pink, grey, and light blue, respectively. The Si atoms are replaced by Al atoms at an Si:Al ratio of 3:1. ***a***, ***b***, and ***c*** are unit vectors for the unit cell of muscovite mica. |***a***| = 5.1579 Å, |***b***| = 8.95 Å, |***c***| = 20.071 Å, *α* = 90.00°, *β* = 95.75°, and *γ* = 90.00°^39^. The model was drawn using VESTA^52^. (**d**) Formation of a thin water film on the muscovite surface. The sample was set in the AFM setup, which was in a Peltier-type thermostatic chamber at 25 °C with a water bath. A small water container was also placed just below the sample to keep the humidity high near the sample. The water vapour gradually condensed on the muscovite surface, resulted in formation of a thin water film. (**e**) AFM imaging of the thin water film on the muscovite surface with an AFM cantilever. Only the tip apex at the end of the cantilever is immersed in the water film. The tip is also covered with a thin water film in humid air.

To examine the solid–water interface on muscovite in humid air, we devised the experimental procedure shown in Fig. 1. A sample of muscovite was cleaved along the (001) plane in laboratory air with an RH of 30–50% (Fig. 1a). An atomistic model of the cleaved muscovite is shown in Fig. 1b and c, from a top view of the (001) plane and a side view of the [010]-directional projection, respectively.

The widely-accepted atomistic model for muscovite mica is as follows^39^: the aluminosilicate layer consists of two hexagonally arrayed sheets of tetrahedrons of (Si,Al)O_2_ and one sheet of octahedrons of Al_2_(O_2_(OH)_2_) between the two tetrahedron sheets. Therein, some O atoms are shared between the tetrahedron sheet and the octahedron sheet. Figure 1b depicts the hexagonal rings, which appear periodically distorted; each ring consists of six O atoms of the upper layer and six Si atoms of the lower layer. The centre of the hexagonal ring is referred to as a ditrigonal cavity, over which a K^+^ ion is located. Al atoms randomly replace the Si atoms in the tetrahedron sheets at an Si:Al ratio of 3:1. On average, two hexagonal rings contain an Al atom in the tetrahedral form with four O atoms with one excess electron. The excess electron provides the tetrahedron sheet with half of a negative unit charge (*e*) per ring. Since the K^+^ ion is located between each cavity of the two facing tetragonal sheets, electrical neutrality around the K^+^ ion holds as + *e* (for K^+^) + 2 × (−1/2 *e* (for the cavity)) = 0. Consequently, the K^+^ layer acts as a weak binder for two facing aluminosilicate layers with negative charge. In Fig. 1b the K^+^ ions on the cleaved surface are depicted randomly positioned over half the cavities of one tetrahedron sheet, although no direct evidence for the K^+^ ion distribution was reported, whereas the existence of K^+^ ions on the surface has previously been revealed using X-ray photoelectron spectroscopy (XPS)^10^.

A sample of cleaved muscovite was set in the FM-AFM setup, which was placed in a thermostatic chamber at 25 °C. A beaker filled with ultrapure water was placed in the chamber, and a small water container was placed just below the sample to keep the humidity high near the sample without forced air-circulation in the chamber. The humidity in the chamber was saturated to be 80% in 2 hours after the setup was prepared. Subsequently, we started to observe the sample by the FM-AFM.

In FM-AFM, the force interaction between the tip and the sample is measured as a change in the resonant frequency (∆*f* ) of the cantilever, which is being oscillated by self excitation of the cantilever (See the detail in Methods). In this study, only the tip apex was immersed in the thin water film (Fig. 1e). Therein, the quality (*Q*) value of the cantilever as an oscillator was not so much reduced in comparison to that for the tip in air. Low values of *Q* value mean that the mechanical oscillation energy is more dissipated, and its resonant characteristics of the oscillator are degraded. The minimum detection limit of the force derivative in this study was 3.4 × 10^−3^ N/m, corresponding to 17 Hz in the unit of frequency (See the detail in Supplementary methods). These values were better by one-order of magnitude than those for the whole cantilever immersed in bulk water, leading to lower *Q* values owing to the mechanical energy dissipation into the water.

### ∆*f* change vs tip-sample distance for the thin water film grown in humid air

**Figure 2:**
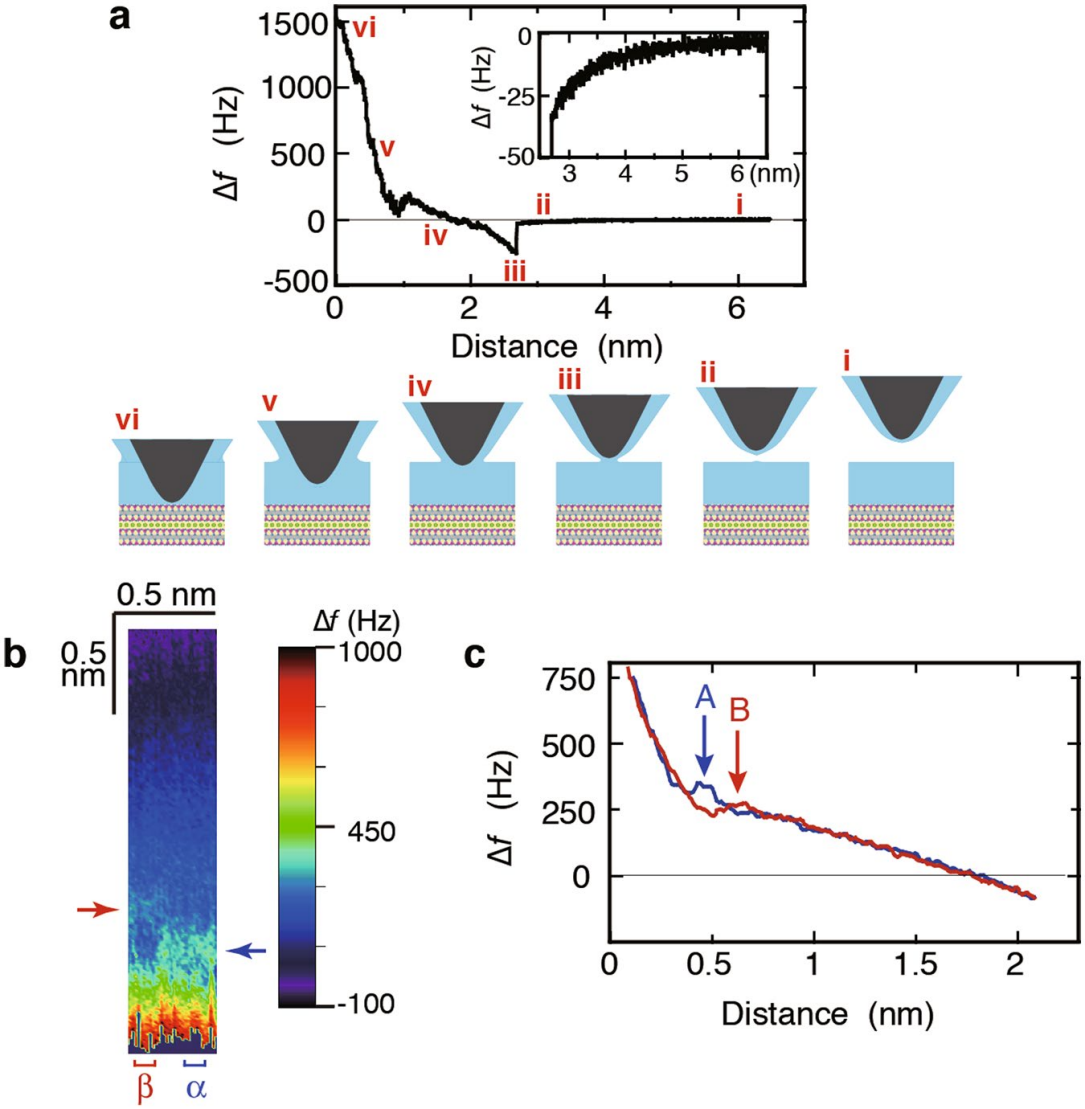
Characteristic changes on ∆*f* with respect to tip–sample distance (*z*). (**a**) Typical ∆*f*–distance (*z*) curve measured for the tip approaching the muscovite surface from above the water film. The schematics of change of the tip distance with respect to the sample are shown from i to vi, corresponding to the tip approach from a far distance to a proximity to the surface. The closest distance was referred to as *z* = 0, shown in vi. This tip distance is regarded as the point of approximate contact between the tip apex and the muscovite surface, at which the cantilever oscillation became unstable and the oscillation amplitude small because of the repulsive forces between the tip and the sample. The inset shows the magnified curve before the tip touched the surface of water film. (**b**) Cutout from a two-dimensional ∆*f* map. The bottom shows the muscovite surface. The tip repeatedly approached and returned in a distance range, e.g., between iv and vi as seen in **a**, while shifting its lateral position step-wise with a ~0.44 nm width of *x*-scan, composed of 33 lines, and a ~2 nm-height of *z*-scan. The tip approach was stopped and the tip retracted when ∆*f* reached 750 Hz. The region with a ∆*f* peak (indicated by the blue arrow) near the surface is denoted by α, and the region with a ∆*f* peak (indicated by the red arrow), about 0.2 nm farther from the surface, is denoted by β. (**c**) ∆*f*–*z* curves, which are extracted from **b** in region α and region β, respectively, labeled by curve A (blue) and curve B (red). Each curve is composed of 8 lines on average. The peak in curve A was found at *z* ≈ 0.4 nm (denoted by arrow A), and the peak in curve B at *z* ≈ 0.6 nm (denoted by arrow B). The resonant frequency, spring constant, and oscillation amplitude of the cantilever were 342.5 kHz, 48 N/m, and 0.46 nm, respectively.

First, we measured ∆*f* as a function of the tip-sample distance (*z*) to establish the thickness of the thin water film (Fig. 2a). The tip approached the muscovite surface through the water film until the strong repulsive forces between the tip and the sample caused the cantilever oscillation to become unstable and the oscillation amplitude to decrease, where the tip was almost in touch with the surface. In Fig. 2a, the closest tip–sample distance was set as *z* = 0, which can be considered to approximately represent the point of contact between the tip apex and the muscovite surface. As the distance decreases from the farthest tip position to *z* = 2.7 nm, ∆*f* gradually becomes more negative (Fig. 2a inset), presumably owing to the weakly attractive van der Waals interactions. At *z* = 2.7 nm, ∆*f* rapidly becomes more negative because of the strong meniscus force between the tip and the sample when the tip apex comes into contact with the water film. At closer distances (i.e., <2.7 nm), ∆*f* gradually increases, which indicates that the thickness of the water film between the tip apex and the muscovite surface was 2.7 nm at the moment of detection of the meniscus force.

It has been reported that under ambient conditions of saturated humidity at 18 °C, muscovite surfaces were covered with approximately 2-nm-thick water films^12^. The additional film thickness of 0.7 nm that we observed could be attributed to the water film that existed on the tip of the AFM cantilever. This film was thinner than the film on the muscovite surface because of surface tension pulling the film over its curved apex towards the tip shank. Thus, the thickness of the water film on the muscovite in the FM-AFM setup was assumed to be approximately 2 nm, comparable to those measured by other methods^12^.

In the close proximity to the muscovite surface in the thin water film, we measured the ∆*f* change with respect to distance *z* while *x*-scanning, in a two-dimensional ∆*f* map (see Methods for details). We often observed a part of checker-board-like patterns in the ∆*f* maps, which were previously reported on the structured hydration layers on surfaces of ionic crystals in bulk water^40^. Figure 2b shows a ∆*f* map with a ~0.44 nm width of *x*-scan and a ~2 nm-height of *z*-scan, where a small number of pieces of the checker-board-like pattern were found near the surface. This indicates that the ∆*f* peaks appeared at different *z* heights (denoted by a red arrow and a blue arrow) in the neighbouring regions for the *x*-scan. We took the average of the ∆*f* changes with respect to distance *z* in regions α and β (Fig. 2b), respectively, composed of the successive eight vertical lines with the respective peaks in ∆*f*. The averaged two curves are shown in Fig. 2c; curve A in blue in region α and curve B in red in region β exhibit a peak at two different *z* (~0.4 nm and ~0.6 nm), respectively, those lacking any other peak, with backgrounds of increase in ∆*f* with decreasing *z*. The characteristics of curve A are very similar to the characteristics reported in refs 31 and 36 using FM-AFM operated in water and molecular dynamics (MD) simulations, respectively. In these reports, the rapid increase in the ∆*f*–*z* curves at *z* < 0.3 nm was attributed to the interaction between the tip and the first hydration layer, and the peak was attributed to the interaction with the second hydration layer. Accordingly, we propose that the site at which curve A in Fig. 2c was acquired was over a cavity in the SiO_2_ tetrahedron sheet.

Because the *z* position of peak B is approximately 0.2 nm farther from the *z* position of peak A, we infer that peak B originated from the interaction with the third hydration layer. Since peaks A and B were not found simultaneously on the same site, curve B was estimated to be acquired over regions except the cavity, e.g., over the hexagonal lattice of the muscovite surface. The separation between peaks A and B is in good agreement with a z-position separation of 0.18 nm between the second and third hydration layers, as calculated by MD simulations for a 3-nm-thick water film on muscovite^20^. According to previous studies used high-resolution X-ray reflectivity experiments and MD simulations for micrometre-thick water films^23, 34^, the third hydration layer seemed 0.2–0.3 nm farther from the second hydration layer and broader than the first and second hydration layers, in their figures, and the water-molecule density in the third hydration layer was close to the density of bulk water; though the authors did not mention these points of the third hydration layer. In addition, MD simulations showed that the separation of peaks corresponding to the second and third hydration layers was larger than 0.3 nm for an approximately 10-nm-thick water film^25, 35^, which was regarded as a thicker water film. By contrast, in this study, the separation between peaks A and B in Fig. 2c was 0.2 nm, which are attributed to the second and third hydration layers, respectively. This implies that the third hydration layer in the thin water film is more tightly structured in closer proximity to the muscovite surface than that in the thicker water film.

### Two-dimensional lateral imaging of the solid–water interface in the thin water film

**Figure 3:**
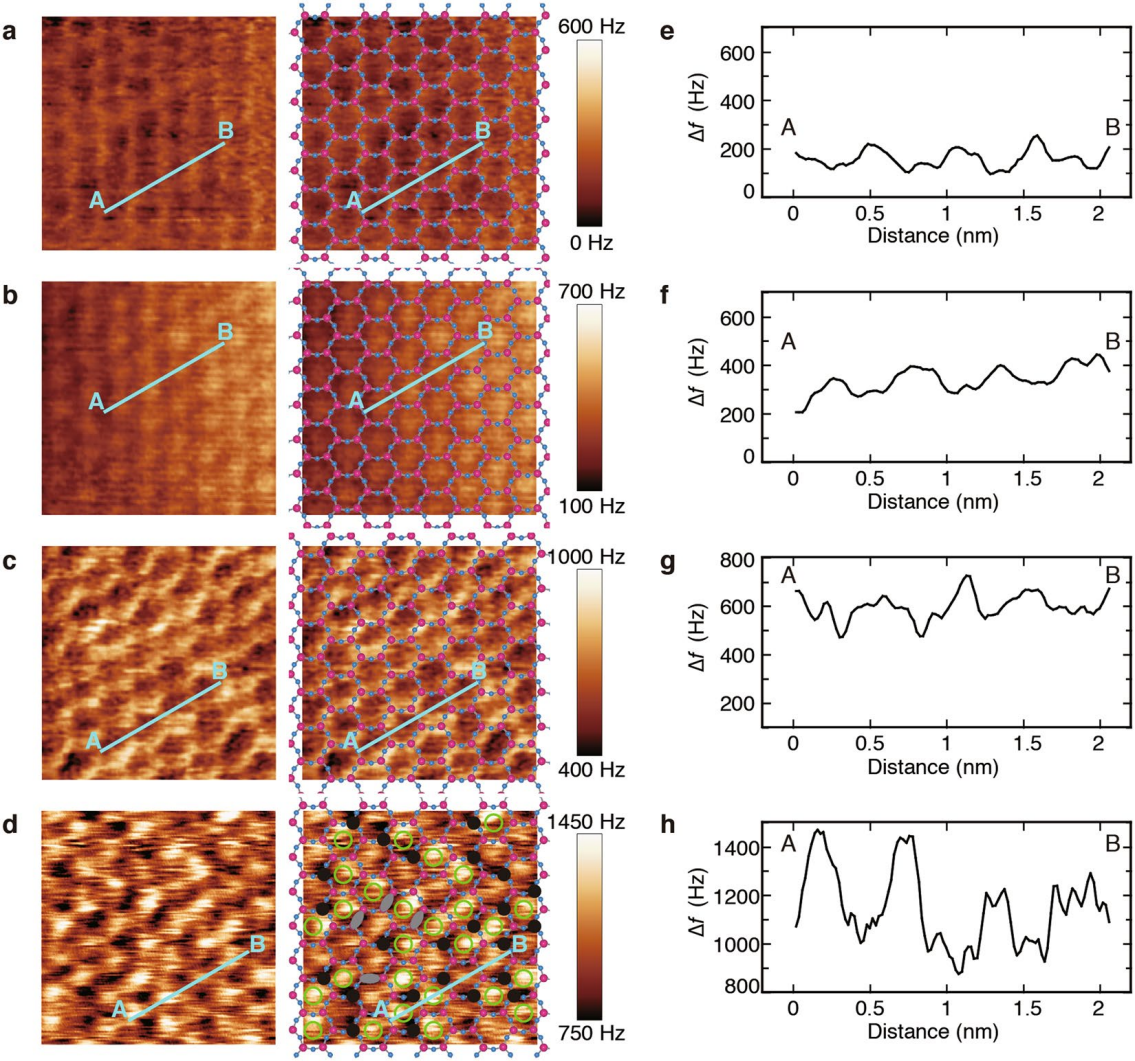
FM-AFM (∆*f* ) images on muscovite surface covered with a thin water film. FM-AFM imaging was conducted in quasi-constant-height mode under weak feedback operation with increasing feedback targets for <∆*f*> of (**a**) 313 Hz, (**b**) 373 Hz, (**c**) 657 Hz, and (**d**) 1044 Hz. The brighter contrast, showing increases in ∆*f* from the averaged target value of ∆*f*, means stronger repulsive force. To highlight the honeycomb structure, in the images on the right, the structural model of the top plane of cleaved muscovite (001) without K atoms is superimposed on the ∆*f* images on the left. Therein, O atoms and Si atoms are shown in blue and pink, respectively. In addition, the contrast features found in **d** are marked on the right panel: the remarkably bright dots at the ditrigonal cavity centres are denoted by open green circles: the dark spots on the Si sites of the hexagonal network by solid black circles; the elongated dark spots by grey ovals. The scan size was 3.5 nm × 3.5 nm. The resonant frequency, spring constant, and oscillation amplitude of the cantilever were 311 kHz, 37 N/m, and 0.5 nm, respectively. The scanning time was 10 sec per image. (**e–h**) ∆*f* cross-sectional profiles along the line between points A and B in **a–d**, respectively. The profiles are parallel to the [110] direction on the muscovite surface. The lines cross over the O atoms and four cavities of the hexagonal framework.

To examine the lateral distribution of water molecules on each structured layer at the solid–water interface, FM-AFM images in Fig. 3a–d were acquired with different feedback targets for ∆*f*, under a quasi-constant-height mode (see Methods). The increase in the targets for ∆*f*, corresponding to the increase in repulsive force between the tip and the sample, indicated that the tip moved progressively closer to the muscovite surface, aiming at the different structured layers. This behaviour of distance change with respect to ∆*f*, seen in Fig. 2c, was commonly observed with different tips. The images in Fig. 3a–d were obtained for the same sample in close succession, though the lateral positions of the images varied due to the thermal drift of the microscope. The brighter regions in the images, showing increases in ∆*f* from the averaged target value of ∆*f*, means that stronger repulsive force acted, and tightly-bound molecules on the surface would be imaged brighter. Faint and bright honeycomb patterns can be observed in Fig. 3a and c, respectively. By contrast, a bright pattern of dots over the cavity centres of the honeycomb lattice is evident in Fig. 3b. A similar dot-arrayed pattern is also evident in the ∆*f* image in Fig. 3d, although the brightness of the dots is non-uniform. The honeycomb patterns and the dot-arrayed patterns were alternately observed in close succession.

Based on appearance of the peaks in the ∆*f*–distance curves in Fig. 2c and previous reports on patterns of AFM images for the hydration layers in much thicker water films^31, 36^, it is most likely that the dot-arrayed pattern in Fig. 3b corresponds to the distribution of water molecules in the second hydration layer (corresponding to peak A), and the honeycomb pattern in Fig. 3c corresponds to the first hydration layer. Namely, in the thin water film, the first hydration layer is shown as a tightly-bound network of water molecules over the honeycomb lattice of the SiO_2_ tetrahedron sheet, and the second hydration layer is shown on the ditrigonal-cavity centres of the tetrahedron sheet, similar to the situation in much thicker water films. The weak honeycomb-pattern contrast in Fig. 3a is also likely to be related to the third hydration layer, as the tip was farther from the second hydration layer (Fig. 3b), although no previous reports have detected the honeycomb structure of the third layer. While Wang *et al*., using MD simulations for a 3-nm thin water film on the muscovite surface, pointed out that the structure of the hydration layer possibly corresponding to our third layer would be related to that of the underneath hydration layer through hydrogen bonding^20^. This means that the structure of the third hydration layer would reflect the periodicity with the muscovite surface under quasi-static conditions. Thus, our findings might indicate that water molecules in the third hydration layer in the thin water film were more tightly bound than in much thicker water films; we name such layers quasi-stabilized hydration layers.

To numerically compare the differences in brightness of ∆*f* in Fig. 3a–d, the cross-sectional profiles along the lines between points A and B are shown in Fig. 3e–h, respectively. Over the non-uniform brightness of the dot-arrayed pattern, in Fig. 3d, the differences in ∆*f* between a peak and its neighbouring valley ranged from about 200 Hz to higher than 300 Hz, in the line profile of Fig. 3h, which were much larger than those in Fig. 3e–g. Because the tip–sample distance was shortest for the highest value of ∆*f* (Fig. 3d), the imaging under the strong repulsive force likely provided a higher resolution to recognize the difference in species, e.g., ions firmly adsorbed on the muscovite surface. In general, K^+^ cations that are adsorbed on the ditrigonal-cavity sites of the muscovite surface can be closely hydrated by water molecules^32, 35^. Thus, the FM-AFM tip detects the repulsive force over the cations more strongly than over the water-adsorbed cavity sites under the quasi-constant-height mode. The remarkably bright dots, denoted by open green circles in the right panel of Fig. 3d, (e.g., the two peaks on the left in Fig. 3h), therefore likely indicate K^+^ ions adsorbed over the ditrigonal-cavity sites, and the bright dots (e.g., the two peaks on the right in Fig. 3h) indicate water molecules adsorbed over the ditrigonal-cavity sites.

Our assignment of the dot-contrast features as K^+^ ions (Fig. 3d) is also supported by the fact that the remarkably bright dots occupied approximately 50% of the total number of cavity sites (precisely, 27 remarkably bright dots with respect to 52 cavity sites). (In addition, we show Figure S1 in Supplementary data with 12 remarkably bright dots with respect to the total number of 21.) This is in agreement with the probability of a K^+^ ion of 50% to be located on either side of the muscovite immediately after cleavage. The other support is the solubility of K^+^ ions in water films. When a muscovite surface is rinsed with pure water, almost all K^+^ ions on the surface are easily removed, and the vacant cavity sites are filled with H_3_O^+^ ions or H_2_O molecules^9, 10, 23^. When a rinsed muscovite surface is immersed in a 5 mM KCl solution, all the cavity sites were filled with K^+^ ions^32^. If all K^+^ ions on the cleaved muscovite surface were to dissolve in a 2-nm-thick water film, the concentration of K^+^ ions would be 1.8 M, which is an extremely concentrated solution. Thus, it seems reasonable that almost all K^+^ ions on the cleaved muscovite surface remain on the surface. Moreover, it is anticipated that equilibrium conditions would be achieved in long time constants after a very small quantity of K^+^ ions on the surface start to be dissolved in the thin water film, because the diffusion velocity of ions on a surface in a thin water film is very slow^10, 18, 19^, resulted in almost unchanged distribution of K^+^ ions on the surface in short time.

In addition, Fig. 3d shows sharp dark spots and a few of elongated dark spots, denoted by solid black circles and grey ovals on the panel, respectively. They are located at the Si (or Al) sites of the tetrahedron sheet which are the crossing points of the honeycomb lattice, and the elongated dark spots seem extending along the honeycomb lattice. The dark spots are possibly ascribed to the negatively-charged Al atoms substituted for Si atoms, because the negatively-charged Al atom can act a stronger attractive force to the tip apex, and lead to a smaller ∆*f* for the darker spots, despite a total repulsive force between the tip and the sample. The numbers of the dark spots and the elongated ones were 24 and 4 in the image, respectively, whereas the total number of the Si (or Al) sites was 98. Accordingly, the Al atoms occupied the Si sites at a rate of 29% (=28/98), including the elongated dark spots counted as one per each. This value is close to the ideal value of 25% (Al:Si = 1:3) to keep the electric neutrality with the K^+^ ions in muscovite. These features were also observed in Figure S1. This faint contrast change has not been reported by using the AFM operated in liquid; the detection ability of our FM-AFM with the high *Q* value would help improving the spatial resolution.

It is of value to reckon the elongated dark spots. If the two Si sites on the elongated dark spot are replaced by Al atoms, the two Al atoms are neighbouring, leading to extra local negative charge. This is not allowed by Al-avoidance rule (Loewenstein’s rule)^41^. Instead, so as to keep the rule for the elongated dark spot, it is possible that the Al atom has the neighbouring defect of a water molecule in the first hydration layer, which should be located on the O site of the hexagonal ring of the tetrahedron sheet. On the other hand, Al-avoidance rule does not hold for some natural minerals^42, 43^; this implies that the rule would not be always applicable. Further study should be undertaken.

As for the ordering of the remarkably bright dots as the K^+^ ions, we counted the number of the dark spots around the bright dots. In Fig. 3d there were 33 hexagon rings having all of 6 sites for Si, and 18 rings were occupied with the K^+^ ions. For the 18 rings, which had dark spots more than one, the numbers of Si and Al per ring were counted as follows; Si_5_Al_1_:Si_4_Al_2_:Si_3_Al_3_ = 7:8:3. On the other hand, for the remaining 15 rings without the K^+^ ions, Si_6_:Si_5_Al_1_:Si_4_Al_2_:Si_3_Al_3_ = 1:4:9:1. Consequently, we found no distinct dependence of the K^+^ ion existence on the number of substituting Al atoms in the ring. For example, for a ring of Si_4_Al_2_, the occupation rate of the K^+^ ion to the ring is ~47% (=8/17), whereas it is ~64% (=7/11) for Si_5_Al_1_. This indicates that electric attractive interactions between the K^+^ ion and the Al atom did not take crucial part in determination of the K^+^ ion distribution. In addition, we found neither ordered nor clustering arrangements of the K^+^ ions with respect to the honeycomb lattice with the dark spots.

**Figure 4:**
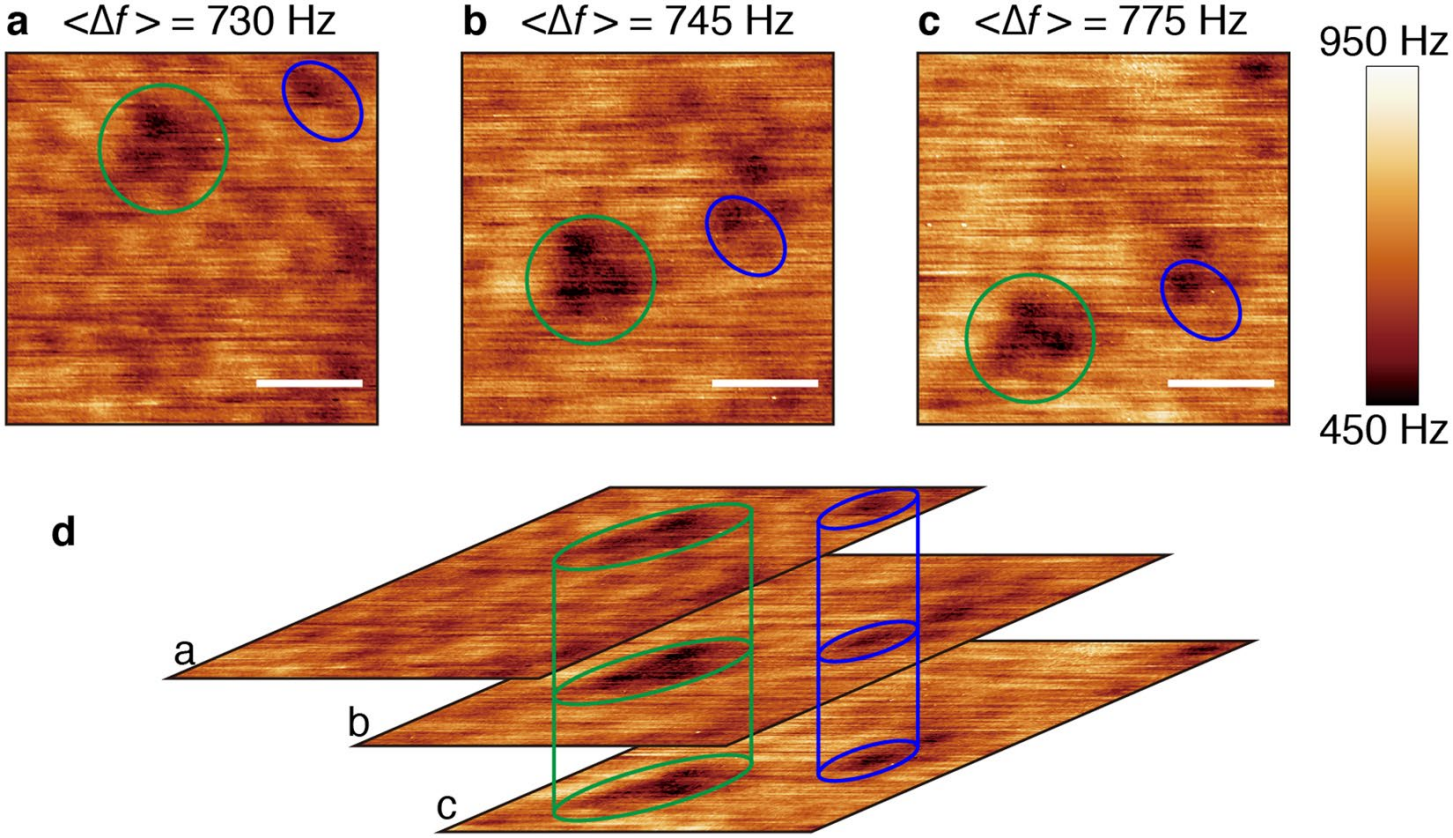
FM-AFM (∆*f*) images on muscovite surface covered with a thin water film with low lateral thermal drift. (**a**) <∆*f*> = 730 Hz. (**b**) <∆*f* > = 745 Hz. (**c**) <∆*f*> = 775 Hz. The green and blue circles mark the same lateral positions with dark contrast on the muscovite surface. The white scale bar represents 1 nm. (**d**) Vertical superimposition of the images in (**a–c**), with the alignment of each of the positions marked by the coloured circles. The dark regions penetrate through multiple water layers. The resonant frequency, spring constant, and oscillation amplitude of the cantilever were 342.5 kHz, 48 N/m, and 0.46 nm, respectively. The scanning time was 15 sec per image. The time intervals between **a** and **b**, and between **b** and **c**, were 114 sec and 53 sec, respectively. The averaged drift velocity was 2.5 pm/s for the *x*-direction, and −11 pm/s for the *y*-direction.

During consecutive FM-AFM image observation in a similar manner to those in Fig. 3, we found the defective structures of water network at the interface. The consecutively-obtained FM-AFM images were shown in Fig. 4a–c. The image obtained at a target ∆*f* value of 730 Hz (Fig. 4a) exhibited a dot-arrayed pattern similar to the pattern shown in Fig. 3b, corresponding to the second hydration layer. The image at 745 Hz (Fig. 4c) corresponds to the first hydration layer with the honeycomb pattern, although the contrast was not as clear as in Fig. 3c, probably because of a slightly greater separation between the tip and the muscovite surface. The image in Fig. 4b was obtained at an intermediate separation, between the ∆*f* values used in Fig. 4a and c. Green and blue circles in each panel indicate discernible dark features that are in the same lateral position on the sample, although they appear in different positions in each panel; despite lower thermal drift in these measurements, these positions slightly shifted in the scanning region. In Fig. 4d, the three images are overlaid for clarity to assign each dark feature, aligning the marked locations. The same dark features can be seen in each image, despite the whole image contrast changing from Fig. 4a to c with increasing ∆*f*.

The above-mentioned dark features in Fig. 4d indicate that defective water-networks formed on the muscovite surface persisted up to the second hydration layer. It is likely that defects in the SiO_2_ tetrahedron sheet of the muscovite surface made it difficult for a network of water molecules (i.e., the hydration layers) to form. Because the thin water film was grown from ambient water vapour and the defects might not act as preferential adsorption sites for water molecules, the water molecules gently and vertically grew into the hydration structures beside the defects. Weakly bound water molecules might cover the defects, resulting in non-uniform hydration layers, which should be imaged dark by the FM-AFM. In Fig. 4d, the vertically-connected dark regions would be evidence for water molecules disordered weakly-bound each other, penetrating from the first to second hydration layers. Fukuma *et al*. observed defects (probably in the first hydration layer) on a cleaved muscovite surface (001) in water using FM-AFM^44^. Siretanu *et al*. reported that the surface defects on smectite of clay mineral were imaged, which were not in the hydration layers^45^. However, at present, there are no reports on the formation of defective regions at the solid–water interface that persist through multiple hydration layers. These regions, which have been observed for the first time in this study, probably arise because layer-by-layer film growth is interrupted over the non-preferential adsorption region for water molecules on the muscovite surface. In other words, this means that the growth of water overlayers in the solid–water interface could proceed in conformation to its underneath water layer through interactions among the water molecules.

## Discussion

Until now, the properties of the solid–water interface has been considered to be irrelevant to the thickness of the water film. However, we have observed that the water structure in thin films grown from ambient water vapour is markedly different from the structure in bulk water. The water network structures can be weakened with increasing thickness of the water film. It is worthwhile to take the entropic effect into account. Individual water molecules as liquid in the water layer of a thick water film possibly have a larger freedom of motion in comparison with that of the two-dimensionally confined water layer of a thin water film. Accordingly, by taking some water molecules in the (third) hydration layers into the water layer of the thick water film, their free energies would be reduced through the increase in entropy due to the freedom change. This may lead to a softened, structured network around the third hydration layer in the thick water film.

This change in free energy of the solid–water interface may be related to hysteresis of the water wettability on the muscovite surface: the water contact angle has been shown to be almost 0° for a muscovite surface that had previously been wet with bulk water^46, 47^, but the contact angle of water has been measured at 6°–7° for a muscovite surface that had been covered with a 2-nm-thick water film grown from ambient water vapour^5, 12^. This indicates that the free energy at the interface between bulk water and the thin water film grown in humid air is temporarily higher than that between bulk water and the thick water film, which would approach the same free energy with a long time constant. The difference in the free energy can affect the reactions through the water films under ambient conditions. Therefore, care of how to prepare the water film should be taken, even if the water film thickness is the same, when interpreting water behaviours on solid surfaces, as their structure and properties can be hysteretically affected by the growth mode.

In summary, we have found that water molecules at the solid–water interface on a muscovite mica surface in a 2-nm-thick water film grown in humid air is more tightly bound each other, differing from ice-like for one-monolayer water adsorption and liquid-like for bulk water. This finding has first been performed using our modified FM-AFM with only the tip apex immersed in the thin water film, giving high *Q* values. Epitaxial-like growth of the thin water film from water vapour of humid air, in forming quasi-stabilized structures of water network, left defective structures at the interface, and the K^+^ ions on the muscovite mica surface. This study suggests that, for the solid–water interfaces where chemical reactions crucially took place, the forming processes of water films, e.g., from dry to wet or vice versa, can alter their properties and reactivity in regard with the atomic and molecular structures of the interfaces.

## Methods

### Sample preparation

The muscovite mica (item No. 990066, Nilaco) sample was cleaved in air along the (001) plane using Scotch tape under ambient laboratory conditions. Immediately after cleavage, the sample was fixed onto a sample holder with leaf springs for the FM-AFM setup. Subsequently, the holder was placed in the FM-AFM setup.

### Frequency-modulation atomic force microscopy (FM-AFM)

In FM-AFM, atomic-level weak forces that act between a sample and a sharp tip in close proximity are detected by measuring a change in the resonant frequency of the cantilever (∆*f* ). The cantilever has a tip at its end and oscillates at its resonant frequency (*f* ). A modified commercial FM-AFM setup (prototype of SPM-8000FM, Shimadzu Corp.)^48, 49^ was used. The FM-AFM setup was equipped with a custom-made, analogue, phase-locked loop with a bandwidth of 1 kHz to achieve low-noise and high-speed FM detection^50^. The amplitude of cantilever oscillation was maintained at approximately 0.5 nm (less than the thickness of the water film). A commercial Si AFM cantilever (ppp-NCHR, Nanosensors) with an Al-coated layer on the back, with a spring constant of 30–50 N/m and a resonant frequency at free (*f*_0_) of 300–350 kHz, was used. The AFM cantilever with a Si tip was cleaned using an ozone cleaner (UV253, Filgen Inc.) just before the measurements to remove hydrocarbon contaminants, making the surface of the tip hydrophilic through the formation of a Si oxide overlayer. The FM-AFM images showing the variation of ∆*f* were acquired in a quasi-constant-height mode with weak feedback from the controller of the FM-AFM setup; the tip detected only the slow variation of the height (*z*) in the surface topography. This FM-AFM system had low-noise detection for the laser-beam deflections used to measure the AFM cantilever displacement. The noise density of the deflection measurements was 15 femtometres/√Hz, which did not change when the ambient humidity was changed or when the tip apex was immersed in the water film. The minimum force detection limit is determined by the *Q* value of the oscillating cantilever (See the detail in Supplementary methods). The *Q* value in air, as measured in this study, ranges from 450 to 600 and is limited by the viscosity of air. When the whole cantilever is immersed in water, the *Q* value is usually reduced to less than 10. However, by immersing only the tip end into the water film, deterioration of the *Q* value can be suppressed; the value was ~200 in this study. Image structuring and analysis of the FM-AFM (∆*f* ) data were performed using the WSxM software (ver. 5)^51^.

### Two-dimensional ∆*f* mapping

To measure the change in ∆*f* with respect to the tip–sample distance, the tip was retracted by approximately 2.2 nm along the *z*-axis from the imaging position after the feedback operation was inactivated, and ∆*f* was measured by the tip approaching from the retracted position to the position where ∆*f* reached a preset value (750 Hz); subsequently, the tip was returned to the retracted position. Next, the position of the tip was slightly shifted along the *x*-axis, and the measurement of the change in ∆*f* with respect to the tip–sample separation was repeated.

### Humidity and temperature control for FM-AFM

The FM-AFM setup was installed in a Peltier-type thermostatic chamber (CN-40A, Mitsubishi Electric Engineering) with a beaker filled with water, and the temperature was maintained at 25 °C. A small container filled with water was placed immediately under the sample, and the upper face of the sample was observed. The relative humidity was measured with a humidity-temperature sensor (HT-3007SD, SATO TECH), placed about 5 cm away from the sample. The value of relative humidity in the chamber saturated above 80% in 1–2 hours after the tip and the sample had been placed into the FM-AFM setup and the hatch of the chamber had been closed.

## Electronic supplementary material

Supplementary Information
