# Supplementary material for: Quasi-stabilized hydration layers on muscovite mica under a thin water film grown from humid air
Source: Sci Rep. 2017 Jun 22;7:4054. doi: 10.1038/s41598-017-04376-3 (PMC5481378; doi:10.1038/s41598-017-04376-3)
Supplement: Supplementary file 1 — Supplementary Information [file 41598_2017_4376_MOESM1_ESM.pdf]

Supplementary Information

Quasi-stabilized hydration layers on muscovite mica under a thin water film grown from humid air

Toyoko Arai[1*], Kohei Sato[1], Asuka Iida[1] & Masahiko Tomitori[2]

[1]Graduate School of Natural Science and Technology, Kanazawa University, Kanazawa, Ishikawa 920-1192, Japan.
[2]School of Materials Science, Japan Advanced Institute of Science and Technology, Nomi, Ishikawa 923-1292, Japan.

Supplementary data

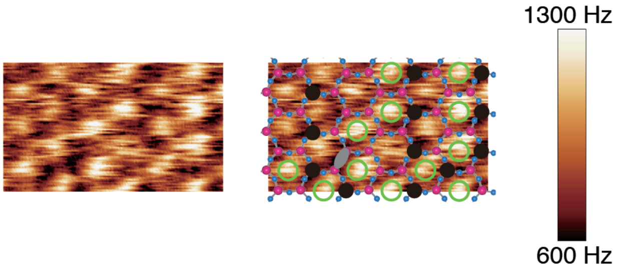

Figure S1 Atom-resolved FM-AFM (Δ*f*) image on muscovite surface covered with a thin water film of 2.5 nm. The K^+ ions are depicted as "*remarkably bright dots"* on the ditrigonal centre sites of the honeycomb lattice of cleaved muscovite (001), and the dark spots possibly correspond to the Al atoms replacing the Si atoms on the hexagonal sites of the honeycomb lattice. To highlight the honeycomb structure, in the image on the right panel, the structural model of the muscovite surface is superimposed, and "*remarkably bright dots"* are denoted by open green circles, and the dark spots are denoted by solid black circles. The number of "*remarkably bright dots*" was 12 with respect to the number of the ditrigonal centre sites of 21 in the image; the K^+ ion occupation rate is ~57%. The number of the dark spots was 12 with respect to the number of the Si atom sites of the honeycomb sites of 46 in the image; the Al atom occupation rate is estimated to be ~28%, which is close to 25% for the ideal occupation rate on the cleaved (001) plane of muscovite in electric charge neutrality with the K^+ ions. FM-AFM imaging was conducted in quasi-constant-height mode under weak feedback operation at a feedback targets for $\langle \Delta f \rangle$ of 896 Hz. The image size was 2.9 nm × 1.7 nm. The resonant frequency, spring constant, and oscillation amplitude of the cantilever were 311 kHz, 37 N/m, and 0.5 nm, respectively.

Supplementary methods

Noise evaluation of frequency modulation atomic force microscopy (FM-AFM) for study of the thin water film on a solid surface

In the FM-AFM the force (F_{ts}) acting between the tip and a sample is detected using a tip-mounted cantilever, being oscillated at its resonant frequency (f). F_{ts} induces a small change of f (the resonant frequency shift (Δf)), which is measured by the FM technique with a phase-locked loop (PLL) circuit. When the oscillation amplitude of the cantilever (A) is as small as a few Å, the relationship between Δf and F_{ts} is expressed as follows, using the force derivative with respect to the tip–sample distance z ($k_{ts} = -dF_{ts}/dz$):

$$\Delta f = \frac{f_0}{2k} k_{ts} . \qquad (1)$$

Here, k is the spring constant of the cantilever, and f_0 is f at $F_{ts} \approx 0$.

The minimum detection limit of Δf is determined by some sorts of noises such as the measurement system noise (N_{def}), the oscillator noise (N_{osc}), and the thermal vibrational noise (N_{th}), which are hereinafter evaluated as noise for measuring k_{ts} by the units of N/m through equation 1. The total noise (N_{total}) can be expressed as follows:[1,2]

$$N_{total} = \sqrt{(N_{def})^2 + (N_{OSC})^2 + (N_{th})^2} = \sqrt{\frac{8k^2 n_d^2 B^3}{3 f_0^2 A^2} + \frac{2k^2 n_d^2 B}{Q^2 A^2} + \frac{4 k k_B T B}{\pi f_0 Q A^2}} , \qquad (2)$$

where n_d is the noise density of the displacement sensor, Q is the quality value of the cantilever of oscillator, B is the bandwidth of the detection system, T is the temperature in Kelvin, and k_B is the Boltzmann constant.

To infer Q and n_d in our FM-AFM setup, thermal vibrational power spectra of the cantilever were measured at relative humidities of 60% and 80% at a large tip−sample distance, and under the conditions of only the tip apex immersed in the thin water film at a small tip–sample distance at a humidity of 80%, as shown in Figure S1. In these measurements the cantilever was in free thermal oscillation, that is, without oscillation excitation. The values of f_0, Q, and n_d were evaluated from the curves in Figure S1. With A = 0.5 nm, k = 31 N/m, and B = 1,000 Hz in this study, the values of N_{def}, N_{OSC}, N_{th}, and N_{total} were calculated, as summarized in Table S1. When the whole cantilever, including the tip, was in air, the values of Q and n_d were almost the same for both humidities of 60% and 80%. For only the tip apex immersed in the thin water film, while the cantilever was in air, n_d did not change, which remained as low as 15 fm/√Hz; a very low noise level was achieved. On one hand, the Q value lowered from 512 to 183, leading to the increases in N_{OSC} and N_{th}. The total noise N_{total} was 3.4×10^{-3} N/m for only the tip apex immersed in the thin water film, which was not degraded so much, compared with 2.0×10^{-3} N/m of N_{total} for the tip apex in air as well as the cantilever.

When the whole cantilever with the same specifications with those of the above-mentioned cantilever, including the tip, is immersed in bulk water, the Q value and f_0 usually turn lower to 5–10 and about 120 kHz, respectively. Assuming that n_d did not change, N_{total} for the

cantilever in bulk water was calculated to be 2.4×10^{-2} N/m, tabulated in Table S1. This indicates that the minimum detection limit of the force derivative with respect to z was deteriorated by one-order of magnitude. In comparison with the performance of FM-AFM with the whole cantilever in bulk water, the performance of the FM-AFM operated in air using only the tip apex immersed in a thin water film is expectedly improved in regard to high sensitivity detection of the force derivative.

Table S1 Q values and noises of the cantilever used in this study measured and evaluated.

Conditions for measurements or evaluations	f_0 [Hz]	Q value	n_d [fm/√Hz]	N_{def} [N/m]	N_{OSC} [N/m]	N_{th} [N/m]	N_{total} [N/m]
in air (RH 60%)	304,930	514	15	1.6x10^{-4}	8.1x10^{-5}	2.0x10^{-3}	2.0x10^{-3}
in air (RH 80%)	304,930	512	15	1.6x10^{-4}	8.1x10^{-5}	2.0x10^{-3}	2.0x10^{-3}
in the water film (RH 80%)	305,060	183	15	1.6x10^{-4}	2.3x10^{-4}	3.4x10^{-3}	3.4x10^{-3}
Assuming that Q = 10, and f_0 lowers to about one third of that in air	(120,000)	(10)	(15)	4.5x10^{-4}	4.7x10^{-3}	2.3x10^{-2}	2.4x10^{-2}

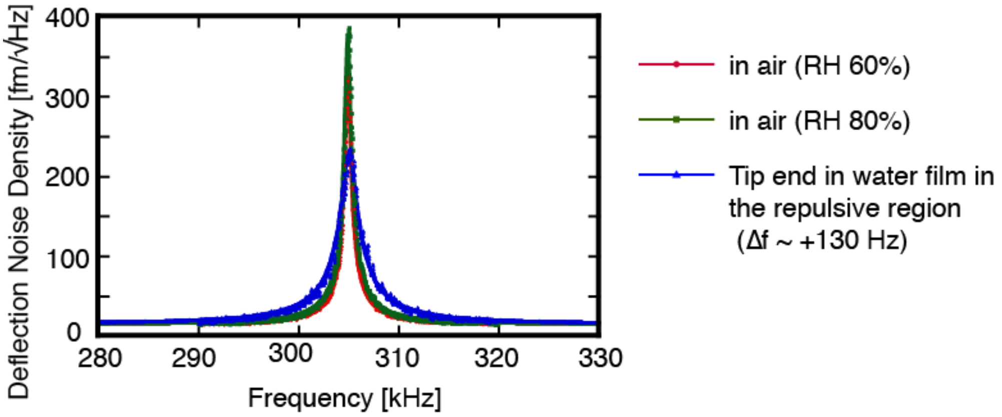

Figure S2 Thermal vibrational power spectra of the cantilever. The red curve was obtained in air with a humidity of 60%, and the green curve was obtained in air with 80%. The blue curve was obtained for only the tip apex immersed in the thin water film on the muscovite surface, whereas the cantilever was in air with a humidity of 80%. The curves were measured at 25 °C.

Supplementary reference

1. Wutscher, E. & Giessibl, F. J. Atomic force microscopy at ambient and liquid conditions with stiff sensors and small amplitudes. *Rev. Sci. Instrum.* **82**, 093703 (2011).
2. Ooe, H., Fujii, M., Tomitori, M. & Arai, T. Evaluation and optimization of quartz resonant-frequency retuned fork force sensors with high Q factors, and the associated electric circuits, for non-contact atomic force microscopy. *Rev. Sci. Instrum.* **87**, 023702 (2016).
